# Universitäre Notaufnahmen in der Coronapandemie – Ergebnisse des *ReCovER-*Registers

**DOI:** 10.1007/s00063-021-00859-4

**Published:** 2021-09-01

**Authors:** Victor Suárez, Felix C. Koehler, Matthias J. Hackl, Martin Möckel, Anna Slagman, Samipa Pudasaini, Joachim Risse, Domagoj Schunk, Sabine Blaschke, Philipp Kümpers, Volker Burst

**Affiliations:** 1 Medizinische Fakultät und Universitätsklinik Köln, Klinische Akut- und Notfallmedizin, Universität zu Köln, Kerpener Str. 62, 50937 Köln, Deutschland; 2 Medizinische Fakultät und Universitätsklinik Köln, Klinik II für Innere Medizin (Nephrologie, Rheumatologie, Diabetes und Allgemeine Innere Medizin) und Zentrum für Molekulare Medizin Köln, Universität zu Köln, Köln, Deutschland; 3 Medizinische Fakultät und Universitätsklinik Köln, CECAD, Universität zu Köln, Köln, Deutschland; 4 Notfall- und Akutmedizin, Zentrale Notaufnahmen, Campus Mitte und Virchow, Charité – Universitätsmedizin Berlin, Berlin, Deutschland; 5 Australian Institute of Tropical Health and Medicine, College of Public Health Medical and Veterinary Sciences, Centre for Chronic Disease Prevention, James Cook University, Cairns, Queensland Australien; 6 Zentrum für Notfallmedizin, Universitätsklinikum Essen, Essen, Deutschland; 7 Interdisziplinäre Notaufnahme, Universitätsklinikum Schleswig-Holstein, Campus Kiel, Kiel, Deutschland; 8 Zentrale Notaufnahme, Universitätsmedizin Göttingen, Göttingen, Deutschland; 9 Medizinische Klinik D, Allgemeine Innere Medizin und Notfallmedizin, Nephrologie und Rheumatologie, Universitätsklinik Münster, Münster, Deutschland

**Keywords:** COVID-19, Notaufnahme, Registerstudie, Deutschland, SARS-CoV‑2, COVID-19, Emergency department, Registry, Germany, SARS-CoV‑2

## Abstract

**Hintergrund:**

Die aktuelle COVID-19-Pandemie stellt, trotz der Verfügbarkeit von Schnelltests und des Starts der Impfkampagne, die Notaufnahme weiterhin vor große Herausforderungen. Die strukturierte Erfassung von demographischen, klinischen sowie therapiebezogenen Daten stellt die Grundlage für die Erstellung evidenzbasierter Prozesse und Behandlungskonzepte dar.

**Ziel der Arbeit:**

Darstellung der systematischen Erfassung klinischer Parameter bei Patienten mit COVID-19-Verdacht im Notaufnahmeregister *ReCovER* (*Registry for COVID-19 in the Emergency Room*) sowie deskriptive Darstellung der ersten 1000 Patienten.

**Material und Methoden:**

In zentralen Notaufnahmen (ZNA) von 6 Universitätskliniken werden kontinuierlich Daten von Patienten mit COVID-19-Verdacht, unabhängig vom Nachweis einer SARS-CoV-2-Infektion, in ein webbasiertes, anonymisiertes Register eingegeben.

**Ergebnisse:**

Zwischen dem 19.05.2020 und dem 13.01.2021 wurden 1000 Patienten in das Register eingegeben, hiervon waren 594 Patienten (59,4 %) in der SARS-CoV-2-positiven (PG) und 406 Patienten (40,6 %) in der negativen Gruppe (NG). Patienten der PG hatten signifikant weniger Vorerkrankungen und eine deutlich längere Latenz zwischen Symptombeginn und Vorstellung in der ZNA (Median 5 vs. 3 Tage), litten häufiger unter Husten, Myalgien, Fatigue sowie Geruchs/Geschmacksverlust und hatten einen signifikant höheren Sauerstoffbedarf als die NG-Patienten. Die Rate von schweren Krankheitsverläufen war in der PG signifikant erhöht und es bestanden nach Entlassung häufiger persistierende Symptome (11,1 vs. 4,6 %).

**Schlussfolgerungen:**

Durch die multizentrische Erfassung von umfangreichen klinischen Daten zu COVID-19-Verdachtsfällen in der Notaufnahme können insbesondere auch für die Situation in Deutschland spezifische Aspekte analysiert werden. Dies ist essenziell für eine gezielte Überprüfung und Adaption international publizierter Strategien.

## Abstract (en)

**Background:**

The current COVID-19 pandemic, despite the availability of rapid tests and the start of the vaccination campaign, continues to pose major challenges to emergency departments (ED). Structured collection of demographic, clinical, as well as treatment-related data provides the basis for establishing evidence-based processes and treatment concepts.

**Aim of the work:**

To present the systematic collection of clinical parameters in patients with suspected COVID-19 in the *Registry for COVID-19 in the Emergency Room *(*ReCovER*) and descriptive presentation of the first 1000 patients.

**Materials and methods:**

Data from patients with suspected COVID-19, regardless of evidence of SARS-CoV‑2 infection, are continuously entered into a web-based, anonymized registry in ED at six university hospitals.

**Results:**

Between 19 May 2020 and 13 January 2021, 1000 patients were entered into the registry, of whom 594 patients (59.4%) were in the SARS-CoV‑2 positive group (PG) and 406 patients (40.6%) were in the negative group (NG). Patients of the PG had significantly fewer pre-existing conditions and a significantly longer latency between symptom onset and presentation to the ED (median 5 vs. 3 days), were more likely to suffer from cough, myalgia, fatigue, and loss of smell/taste and had significantly higher oxygen requirements than NG patients. The rate of severe disease progression was significantly higher in the PG, and persistent symptoms were more common after discharge (11.1 vs. 4.6%).

**Conclusions:**

The multicenter collection of comprehensive clinical data on COVID-19 suspected cases in the ED allows analysis of aspects specific to the situation in Germany in particular. This is essential for a targeted review and adaptation of internationally published strategies.

## Einleitung

Seit Beginn des Jahrs 2020 besteht eine weltweite Pandemie durch das SARS-CoV-2-Virus [1, 5]. Das Spektrum von COVID-19 reicht von einfachen Erkältungssymptomen bis zum beatmungspflichtigen Lungenversagen (Acute Respiratory Distress Syndrome, ARDS; [10]). Notaufnahmen sind oft der erste Anlaufpunkt bei entsprechenden Beschwerden, was regional die Kapazitäten der Notaufnahmen und der Intensivstationen stark belastet und teilweise zu erheblichen Kapazitätsengpässen führt [3, 10]. Das Personal der Notaufnahmen hat eine Schlüsselrolle, da es neben der Erstversorgung auch für die Entscheidung über die primär ambulante oder stationäre Behandlung zuständig ist [6, 8]. Um Daten für den deutschen Raum besser zu erfassen und strukturiert auszuwerten, wurde im Mai 2020 das Notaufnahmeregister *ReCovER* (*Registry for COVID-19 in the Emergency Room*) installiert, an dem sich aktuell 6 universitäre Notaufnahmen in Deutschland kooperativ beteiligen. Primäres Ziel dabei war es, Patienten mit einer nachgewiesenen SARS-CoV-2-Infektion, die sich mit einem klinischen Verdachtsmoment, also entsprechenden Symptomen, in der Notaufnahme vorstellen, zu erfassen und zu charakterisieren. Asymptomatische COVID-19-Patienten oder Patienten, die z. B. vor Aufnahme in das Krankenhaus im Rahmen eines in der Notaufnahme durchgeführten Screeningtests als infiziert erkannt wurden, wurden explizit ausgeschlossen, um eine Verwässerung zu verhindern. Zur besseren Beschreibung der Kohorte wurde außerdem eine Referenzgruppe von Patienten in das Register mit aufgenommen, die sich ebenfalls mit COVID-19-typischen Symptomen, jedoch ohne SARS-CoV-2-Nachweis in den Notaufnahmen vorstellten.

### Ziel der Arbeit

Das Ziel dieser Arbeit ist die Darstellung des *ReCovER*-Registers (Aufbau, Protokoll) zur systematischen Erfassung klinischer Parameter bei Patienten mit COVID-19-Verdacht in der Notaufnahme sowie die Beschreibung der ersten 1000 Patienten.

## Methodik

### Studiendesign

**Figure 1:**
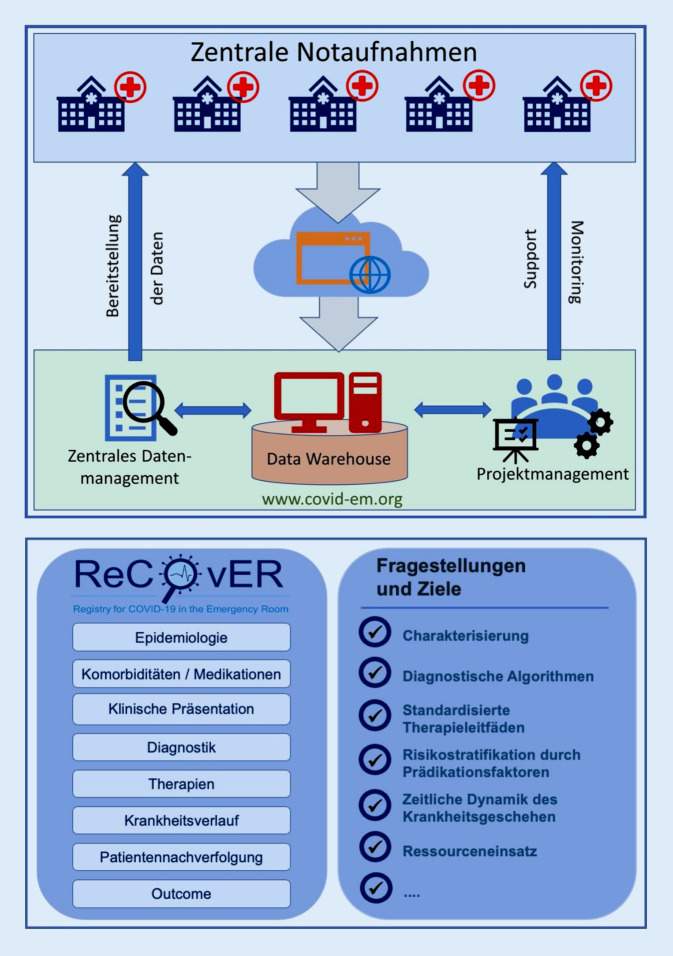

*ReCovER* ist ein offenes, webbasiertes Register, das die anonymisierte, retrospektive Eingabe von Daten zu Patienten aus der Notaufnahme mit Verdacht auf COVID-19 ermöglicht. Nationale und EU-weite Anforderungen an den Datenschutz werden erfüllt. Auf Grundlage der Anonymisierung ist eine Aufklärung und Einwilligung der Patienten nicht notwendig [11]. Die Eingabemaske enthält mehr als 800 Items zu Epidemiologie, Vorerkrankungen, Symptomen sowie Monitordaten bei Vorstellung. Zusätzlich werden die diagnostischen Ergebnisse inklusive der Laborergebnisse und Bildgebung integriert. Ebenso bildet das Register den gesamten klinischen Verlauf bis zur Entlassung, entweder direkt aus der Notaufnahme oder erst nach einem stationären Aufenthalt, ab. Im Rahmen der Pandemie wurden und werden vielerorts betroffene Patienten im Sinne einer Qualitätssicherung auch nach Entlassung nachverfolgt – auch diese Daten können in *ReCovER* abgebildet werden. Für die schnelle, aber dennoch strukturierte Dateneingabe benutzt *ReCovER* ein modulares Design, das Fragen und Items entsprechend dem Krankheitsverlauf anzeigt (Abb. 1).

Die Zustimmung der lokalen Ethikkommissionen (EK) wurde an allen Standorten eingeholt (federführende EK: Ethikkommission der Medizinischen Fakultät der Universität zu Köln, Az.: 20-1198), und die Studie wird nach den Vorgaben der Deklaration von Helsinki und den Richtlinien über gute klinische Praxis (GCP) durchgeführt (*International Conference on Harmonization*). Die Studie ist unter *clinicaltrials.gov* registriert (Identifier: NCT04351854).

### Datensammlung

Aktuell erfassen 6 universitäre Notaufnahmen in Deutschland kontinuierlich Daten in *ReCovER*: Universitätsklinik Köln; Charité – Universitätsmedizin Berlin, Campus Mitte und Virchow; Universitätsklinikum Essen; Universitätsklinikum Schleswig-Holstein, Campus Kiel; Universitätsmedizin Göttingen und Universitätsklinikum Münster. Eingeschlossen werden alle Patienten mit einem positiven SARS-CoV-2-Nachweis im nasopharyngealen Abstrich (PCR), die sich mit mindestens einem COVID-19-verdächtigen Symptom vorstellten, diese Gruppe wird im Folgenden als Positivgruppe (PG) bezeichnet. Als COVID-19-verdächtige Symptome gelten entsprechend der Handreichungen des Robert Koch-Instituts (RKI): Fieber, Husten, Kurzatmigkeit, Myalgien, Fatigue, Geruchs- oder Geschmacksverlust, Diarrhöen, Übelkeit und Erbrechen. Patienten ohne diese Symptome werden – unabhängig vom Ergebnis einer SARS-CoV-2-Testung – nicht eingeschlossen. Um eine bessere Charakterisierung der Patienten in der PG zu ermöglichen, werden darüber hinaus Patienten mit negativem SARS-CoV-2-PCR-Test, aber mit mindestens einem COVID-19-verdächtigen Symptom als Referenzgruppe eingeschlossen, diese Gruppe wird im Folgenden als Negativgruppe (NG) bezeichnet. Bei der Auswahl dieser Patienten war ein „*matching*“ im Sinne einer klassischen Fall-Kontroll-Strategie aufgrund der vor allem zu Beginn der Pandemie beobachteten geringen Notaufnahmefrequentierung nicht möglich. Um das Risiko eines Selektionsbias zu minimieren, wird zu jedem Patienten aus der PG derjenige SARS-CoV-2-negative, symptomatische Patient mit dem zeitlich am nächsten gelegenen Aufnahmezeitpunkt in die NG aufgenommen.

Die Dateneingabe erfolgt frühestens 14 Tage nach Entlassung des Patienten aus dem Krankenhaus, um eine Patientennachverfolgung in diesem Zeitraum zu ermöglichen – erneute Vorstellungen in der Notaufnahme, stationäre Aufnahmen, aber auch Informationen aus in der dieser Zeit dokumentierten telefonischen Kontakten oder ambulanten Vorstellungen können so ebenfalls erfasst werden.

### Statistik

Die deskriptive Statistik wurde mittels SPSS Software Version 26 (IBM Corp., Armonk, NY, USA) durchgeführt. Kategorische Variablen an Patientencharakteristika wurden mittels Frequenz und prozentualem Anteil zusammengefasst sowie mittels χ^2^-Test oder Fishers exaktem Test verglichen. Kontinuierliche Variablen wurden mittels des Medians und des Interquartilsabstands (IQR) zusammengefasst und mithilfe des Mann-Whitney-U-Tests (als Test für unverbundene Stichproben) verglichen. Aufgrund des explorativen Charakters der Analyse wurde nicht für multiples Testen korrigiert, ein *p*-Wert unter 0,05 wurde als statistisch signifikant interpretiert.

## Ergebnisse

Eingaben in *ReCovER* waren ab dem 19.05.2020 möglich. Die hier gezeigte Auswertung bezieht sich auf die ersten 1000 vollständig eingegeben Patienten, dieser Meilenstein wurde am 13.01.2021 erreicht. Ein Zentrum konnte nur Patienten aktiv beisteuern, die nach September 2020 vorstellig wurden, bei allen anderen Zentren wurden Patienten seit Beginn der Pandemie eingeschlossen. In der PG wurden 594 Patienten (59,4 %), in der NG 406 Patienten (40,6 %) eingegeben. Die unterschiedlichen Gruppengrößen ergeben sich aufgrund der lokal unterschiedlichen Eingabepraktiken. Die 594 Patienten in der PG stellen ca. 90 % aller bis zum 13.01.2021 behandelten und somit prinzipiell einschließbaren Patienten dar.

Die Altersverteilung zeigt in beiden Gruppen keine signifikanten Unterschiede, die meisten Patienten in beiden Gruppen waren zwischen 30 und 49 Jahren alt; auch die Verteilung der Geschlechter unterscheidet sich in beiden Gruppen nicht. Patienten der PG litten deutlich seltener an Vorerkrankungen im Vergleich zur NG (28,3 vs. 18,7 %, *p* = 0,001). Vor allem hämatoonkologische Vorerkrankungen (11,8 vs. 20,4 %, *p* < 0,001) sowie chronische Lungenerkrankungen (11,3 vs. 19 %, *p* = 0,001) waren seltener als in der NG (Tab. 1).

**Table 1:** 

	SARS-CoV‑2 positiv (PG)	SARS-CoV‑2 negativ (NG)	*p*-Wert
*Eingeschlossene Fälle gesamt*	*594*	*406*	*–*
**Altersgruppen (Jahre),** ***n*** **(%)**			
< 18	6 (1,0)	4 (1,0)	1,000
18–29	72 (12,1)	54 (13,3)	0,581
30–49	167 (28,1)	97 (23,4)	0,137
50–64	147 (24,7)	93 (22,9)	0,503
65–74	82 (13,8)	64 (15,8)	0,389
75–89	104 (17,5)	84 (20,7)	0,217
≥ 90	16 (2,7)	10 (2,5)	0,822
**Geschlecht,** ***n*** **(%)**			
Männlich	339 (57,1)	225 (55,4)	0,604
**Vorerkrankungen,** ***n*** **(%)**^a^			
Hämato-/onkologische Erkrankung	70 (11,8)	83 (20,4)	**<** **0,001**
HIV/AIDS	5 (0,8)	4 (1,0)	0,992
Organtransplantation	14 (2,4)	10 (2,5)	0,895
Chronische Herzerkrankung	231 (38,9)	176 (43,3)	0,159
Chronische Lebererkrankung	19 (3,2)	18 (4,4)	0,310
Chronische Lungenerkrankung	67 (11,3)	77 (19,0)	**0,001**
Chronische Nierenerkrankung	62 (10,4)	50 (12,3)	0,355
Rheumatologische/Autoimmunerkrankung	31 (5,2)	21 (5,2)	0,974
Diabetes mellitus	108 (18,2)	62 (15,3)	0,238
Über- oder Untergewicht^b^	71 (12,0)	39 (9,6)	0,244
Keine Vorerkrankungen	168 (28,3)	76 (18,7)	**0,001**

*AIDS* „acquired immune deficiency syndrome“, *HIV* „human immunodeficiency virus“, *NG* Negativgruppe, *PG* Positivgruppe, *RT-PCR* „real-time polymerase chain reaction“, *SARS-CoV‑2* „severe acute respiratory coronavirus 2“

^a^> 1 Antwort möglich pro Patient

^b^Übergewicht: Body mass index (BMI) > 30 kg/m^2^, Untergewicht: BMI < 18,5 kg/m^2^

Die Verteilung auf die Dringlichkeitskategorien in den entsprechend der gesetzlichen Vorgaben in allen Zentren durchgeführten Triagesystemen (Tab. 2) zeigt ein klares Übergewicht bei den PG-Patienten in der Kategorie 4 (39,4 vs. 32 %, *p* = 0,017), während NG-Patienten vermehrt in die Kategorie 3 eingeteilt wurden (40,9 vs. 30 %, *p* < 0,001). Es sei angemerkt, dass ein Zentrum den *Emergency Severity Index, *alle anderen Zentren das *Manchester Triage System* benutzen, was gewisse Einschränkungen der Vergleichbarkeit mit sich bringt. COVID-19-Patienten wiesen im Durchschnitt eine deutlich längere Latenz zwischen Beginn der Symptome und Vorstellung in der Notaufnahme auf (5 vs. 3 Tage, *p* < 0,001).

**Table 2:** 

	SARS-CoV‑2 positiv (PG)	SARS-CoV‑2 negativ (NG)	*p*-Wert
**Triage,** ***n*** **(%)**	532	384	–
Kategorie 1	13 (2,2)	8 (2,0)	0,813
Kategorie 2	71 (12,0)	64 (15,8)	0,078
Kategorie 3	178 (30,0)	166 (40,9)	**<** **0,001**
Kategorie 4	234 (39,4)	130 (32,0)	**0,017**
Kategorie 5	36 (6,1)	16 (3,9)	0,138
**Vitalzeichen, Median (IQR)**			
*Auswertbare Fälle*	540	383	**–**
Herzfrequenz (/min)	89 (78–102)	92 (80–106)	**0,019**
Systolischer Blutdruck (mm Hg)	130 (118–141)	134 (119–146)	**0,013**
Diastolischer Blutdruck (mm Hg)	80 (71–89)	80 (70–90)	0,897
Temperatur (°C)	37,1 (36,5–38,0)	37,0 (36,5–38,0)	0,873
SO_2_ (%)	97 (95–99)	98 (95–99)	0,362
qSOFA ≥ 2, *n* (%)	31 (5,2)	13 (3,2)	0,127
Beschwerdebeginn vor Vorstellung, Tage (IQR)	5 (3–8)	3 (2–7)	< **0,001**
**Klinische Symptome, *n* (%)**^**a**^			
*Auswertbare Fälle*	594	406	**–**
Fieber	330 (55,4)	215 (53,0)	0,442
Husten	313 (52,5)	179 (44,1)	**0,008**
Tachykardie	149 (25,0)	142 (35,0)	**0,001**
Geschmacksverlust	85 (14,3)	15 (3,7)	**<** **0,001**
Geruchsverlust	57 (9,6)	12 (3,0)	**<** **0,001**
Diarrhöen	36 (6,0)	29 (7,1)	0,495
Übelkeit/Erbrechen	39 (6,5)	17 (4,2)	0,108
Myalgien	116 (19,5)	49 (12,1)	**0,002**
Fatigue	221 (37,1)	112 (27,6)	**0,002**
Kopfschmerzen	97 (16,3)	54 (13,3)	0,189
Verwirrtheit	9 (1,5)	11 (2,7)	0185
Bewusstseinseinschränkung	9 (1,5)	12 (3,0)	0,119

Die bei der Triage angegebenen Kategorien geben die Klassifikation in die 5 erfassten Stufen des jeweiligen Systems (5-mal Manchester Triage System, einmal Emergency Severity Index) wider

*NG* Negativgruppe, *PG* Positivgruppe, *qSOFA* „quick sepsis-related organ failure assessment“, *S*_*p*_*O2* pulsoxymetrisch gemessene Sauerstoffsättigung

^a^> 1 Antwort möglich

Patienten der PG hatten in der ZNA eine niedrigere Herzfrequenz (89 vs. 92/min, *p* = 0,019), einen niedrigeren systolischen Blutdruck (130 vs. 134 mm Hg, *p* = 0,013) sowie eine höhere Atemfrequenz (18 vs. 16/min, *p* = 0,018) als in der NG. Der Anteil der Patienten mit einem qSOFA-Score ≥ 2 unterschied sich zwischen den beiden Gruppen nicht (5,2 vs. 3,2 %, *p* = 0,127).

In der Analyse der klinischen Zeichen bei Vorstellung waren in der PG vor allem Husten, Myalgien, Fatigue sowie Geruchs- und Geschmacksverlust signifikant erhöht gegenüber der NG. Patienten der NG wiesen dagegen häufiger Tachykardien mit einer Herzfrequenz > 100/min auf (Tab. 2).

Die Analyse der Laborwerte zeigte bei PG-Patienten gegenüber den NG-Patienten erhöhte Werte für Laktatdehydrogenase (LDH), Ferritin, Aspartat-Aminotransferase (ASAT), Alanin-Aminotransferase (ALAT) und Hämoglobin (Hb). Gegenüber den NG-Patienten erniedrigt waren hingegen die Lymphozyten- und Thrombozytenzahlen, das C‑reaktive Protein (CRP) und das Prokalzitonin (PCT; Tab. 3).

**Table 3:** 

	SARS-CoV‑2 positiv (PG)	SARS-CoV‑2 negativ (NG)	*p*-Wert
*Eingeschlossene Fälle gesamt*	594	406	**–**
**Laborergebnisse, Median (IQR)**			
Leukozyten × 10^9^/ml (*n*_miss_ = 150)	6,1 (4,7–8,4)	9,0 (6,4–13,1)	**<** **0,001**
Lymphozyten × 10^9^/ml (*n*_miss_ = 558)	1,0 (0,7–1,5)	1,3 (0,8–2,0)	**0,001**
Thrombozyten × 10^9^/ml (*n*_miss_ = 196)	191 (150–254)	232 (178–291)	**<** **0,001**
Hämoglobin (g/dl, *n*_miss_ = 197)	13,5 (12,3–14,6)	12,5 (11,1–14,4)	**<** **0,001**
C‑reaktives Protein (mg/dl, *n*_miss_ = 171)	7,4 (2,4–23,8)	8,0 (2,5–25,2)	**<** **0,001**
Prokalzitonin (µg/l, *n*_miss_ = 473)	0,10 (0,07–0,20)	0,15 (0,09–0,47)	**<** **0,001**
Laktatdehydrogenase (U/l, *n*_miss_ = 279)	291 (228–403)	249 (206–319)	**<** **0,001**
Ferritin (µg/l, *n*_miss_ = 675)	521 (148–1093)	190 (88–520)	**<** **0,001**
Alanin-Aminotransferase (U/l, *n*_miss_ = 333)	29 (19–45)	24 (17–41)	**0,015**
Aspartat-Aminotransferase (U/l, *n*_miss_ = 377)	38 (28–58)	27 (21–43)	**<** **0,001**
Kreatinin (mg/dl, *n*_miss_ = 177)	0,95 (0,77–1,20)	0,94 (0,75–1,22)	0,864
Natrium (mmol/l, *n*_mis_s = 218)	138 (135–140)	138 (135–141)	0,375
Kalium (mmol/l, *n*_miss_ = 219)	4,0 (3,7–4,3)	4,0 (3,7–4,4)	0,162

*IQR* Interquartilsabstand, *NG* Negativgruppe, *PG* Positivgruppe

In der initialen CT-Bildgebung zeigten sich in der PG häufiger multiple und überwiegend peripher lokalisierte Läsionen. Auch Milchglasinfiltrate und das „*Crazy*-*paving“-*Muster konnte häufiger bei PG-Patienten nachgewiesen werden. Eine Lungenultraschalluntersuchung kam nur selten zur Anwendung (Tab. 4).

**Table 4:** 

	SARS-CoV‑2 positiv (PG)	SARS-CoV‑2 negativ (NG)	*p*-Wert
*Eingeschlossene Fälle gesamt*	**594**	**406**	**–**
**Thorax-CT,** ***n*** **(%)**^a^	200 (33,7)	137 (33,7)	0,981
Singuläres Infiltrat	2 (1,0)	8 (5,8)	**0,018**
Multiple Infiltrate	95 (47,5)	35 (25,5)	**<** **0,001**
Periphere Infiltrate	99 (49,5)	33 (24,1)	**<** **0,001**
Zentrales Infiltrat	4 (2,0)	22 (16,1)	**<** **0,001**
Milchglasinfiltrate	153 (76,5)	47 (34,3)	**<** **0,001**
„Crazy paving“	45 (22,5)	5 (3,6)	**<** **0,001**
Pleuraerguss	13 (6,5)	25 (18,2)	**0,001**
Lungenarterienembolie	4 (2,0)	0	0,149

*CT* Computertomographie, *NG* Negativgruppe, *PG* Positivgruppe

^a^> 1 Antwort möglich pro Patient

Patienten der PG erhielten in der Notaufnahme häufiger Sauerstoff, während sich die Rate an nichtinvasiven und invasiven Beatmungen in beiden Gruppen nicht unterschied. Außerdem erhielten PG-Patienten in der Notaufnahme seltener eine medikamentöse Therapie, insbesondere weniger Antibiotika, Virostatika und Kortison. Auch die Behandlung mit Antiobstruktiva, Antihypertensiva und Diuretika war bei PG-Patienten signifikant seltener zu beobachten (Tab. 5).

**Table 5:** 

	SARS-CoV‑2 positiv (PG)	SARS-CoV‑2 negativ (NG)	*p*-Wert
*Eingeschlossene Fälle gesamt*	594	406	–
**Behandlung in der Notaufnahme, *n* (%)**^**a**^			
Katecholamintherapie	3 (0,5)	5 (1,2)	0,281
Sauerstoffgabe	86 (14,5)	39 (9,6)	**0,022**
High-flow-Sauerstoff (HFNC)	19 (3,2)	14 (3,4)	0,828
Nichtinvasive Beatmung (NIV)	4 (0,7)	4 (1,0)	0,722
Invasive Beatmung	6 (1,0)	2 (0,5)	0,484
Volumensubstitution	203 (34,2)	134 (33,0)	0,701
Pharmakotherapie	236 (39,7)	230 (56,7)	**<** **0,001**
Virostatika	3 (0,5)	18 (4,4)	**<** **0,001**
Antibiotika	70 (11,8)	126 (31,0)	**<** **0,001**
Glukokortikoide	9 (1,5)	17 (4,2)	**0,009**
Antihypertensiva	6 (1,0)	11 (2,7)	**0,041**
Pulmonale Antiobstruktiva	13 (2,2)	31 (7,6)	**<** **0,001**
Diuretika	2 (0,3)	18 (4,4)	**<** **0,001**
Antipyretika	44 (7,4)	23 (5,7)	0,279

*HFNC* „high flow nasal cannula“, *NG* Negativgruppe, *NIV* „non-invasive ventilation“, *PG* Positivgruppe

^a^> 1 Antwort möglich

Während sich die Entlassungsraten unmittelbar aus der Notaufnahme in beiden Gruppen nicht signifikant unterschieden, zeigten Follow-up-Untersuchungen, dass PG-Patienten nach Entlassung häufiger persistierende Symptome (11,1 vs. 4,6 %, *p* = 0,018) hatten und bei erneuter Vorstellung häufiger stationär aufgenommen werden mussten (6,8 vs. 2,3 %, *p* = 0,035; Tab. 6). Die Hospitalisierungsraten für Normal- oder Intensivstationen unterschieden sich in den Gruppen nicht, allerdings war die Dauer sowohl des normalstationären Aufenthalts als auch auf der Intensivstation bei PG-Patienten deutlich erhöht (Median: 9 vs. 7 Tage, *p* = 0,001 auf der Normalstation bzw. 8 vs. 4 Tage, *p* = 0,001 auf der Intensivstation). Der stationäre Verlauf unterschied sich in den Gruppen vor allem durch eine höhere Rate an viralen Pneumonien (68,0 vs. 13,0 %, *p* < 0,001) mit beatmungspflichtigem Lungenversagen (ARDS) in der PG-Gruppe (5,8 vs. 1,3 %, *p* = 0,006). Auch schwere Krankheitsverläufe, definiert als Tod, kardiopulmonale Reanimation (CPR), Intensivaufenthalt, High-flow-Sauerstoffgabe (HFNC), nichtinvasive oder invasive Beatmung, Katecholamintherapie, Sepsis, disseminierte intravasale Gerinnung oder Hospitalisierung > 14 Tage fanden sich häufiger in der PG-Gruppe (26,3 vs. 20,4 %, *p* = 0,034).

**Table 6:** 

	SARS-CoV‑2 positiv (PG)	SARS-CoV‑2 negativ (NG)	*p*-Wert
*Eingeschlossene Fälle gesamt*	594	406	–
Entlassung aus der Notaufnahme, n (%)	235 (39,6)	175 (43,1)	0,264
Erneute Vorstellung in der Notaufnahme	23 (9,8)	10 (5,7)	0,134
Stat. Aufnahme nach erneuter Vorstellung in der Notaufnahme	16 (6,8)	4 (2,3)	**0,035**
Persistierende Symptome nach Entlassung	26 (11,1)	8 (4,6)	**0,018**
Aufnahme auf Normalstation aus der Notaufnahme, n (%)	297 (50,0)	194 (47,8)	0,491
Dauer der Hospitalisierung (Tage), Median (IQR; *n*_miss_ = 10)	9 (5–14)	7 (4–11)	**0,001**
Aufnahme auf Intensivstation aus der Notaufnahme, n (%)	62 (10,4)	37 (9,1)	0,491
Dauer der Hospitalisierung (Tage), Median (IQR; *n*_miss_ = 25)	8 (4–14)	4 (3–6)	**0,001**
**Krankheitsverlauf bei hospitalisierten Patienten, *n* (%)**^**a**^			
Akute virale Pneumonie	244 (68,0)	30 (13,0)	**<** **0,001**
ARDS	21 (5,8)	3 (1,3)	**0,006**
Arrhythmien	4 (1,1)	9 (3,9)	**0,025**
Sepsis	17 (4,7)	16 (6,9)	0,258
Schwerer Krankheitsverlauf^b^	156 (26,3)	83 (20,4)	**0,034**
Tod, n (%)	53 (8,9)	23 (5,7)	0,056
Tod während stat. Aufenthalt	47 (7,9)	20 (4,9)	0,064
Tod in der Notaufnahme	6 (1,0)	3 (0,7)	0,746

*ARDS* Acute Respiratory Distress Syndrome, *IQR* Interquartilsabstand, *NG* Negativgruppe, *PG* Positivgruppe

^a^> 1 Antwort möglich

^b^Schwerer Krankheitsverlauf definiert als: Tod, kardiopulmonale Wiederbelebung (CPR), Aufnahme auf eine Intensivstation, Therapie mit high flow nasal cannula (HFNC), nichtinvasiver Beatmung (NIV) oder invasiver Beatmung, Kreislaufunterstützung, Sepsis, Massenblutung/Disseminierte intravasale Gerinnung oder Hospitalisierung > 14 Tage

## Diskussion

Die COVID-19-Pandemie hat zu intensiver Forschung geführt und dazu beigetragen, das Krankheitsbild in kurzer Zeit umfassend zu charakterisieren [4, 7, 10, 13]. Dennoch ist es bisher nicht gelungen, für die initiale Phase der klinischen Notfallversorgung hinreichend validierte Prädiktionsfaktoren für einen komplizierten Verlauf zu finden, da in den meisten klinischen Beobachtungsstudien eine ausreichend lange Nachbeobachtung der Patienten fehlt [12]. Die Struktur von *ReCovER* adressiert dies, indem Patientendaten grundsätzlich erst mit einer zeitlichen Latenz eingegeben werden, um den außerklinischen Verlauf der Patienten abbilden zu können. Darüber hinaus ermöglicht der Einschluss von SARS-CoV-2-negativen Verdachtsfällen die Analyse attributiver klinischer Merkmale von SARS-CoV-2-positven Patienten in der ZNA. Die Charakterisierung der ersten 1000 Patienten aus *ReCovER* zeigt, dass das Bild an 6 deutschen Universitätskliniken erheblich von dem abweicht, was wir aus der Berichterstattung aus Regionen wie der Lombardei (Italien), New York (USA) oder Lissabon (Portugal) kennen. So handelt es sich in unserer Analyse vor allem um Patienten mittleren Lebensalters, die bei Vorstellung in der Mehrzahl der Fälle nicht schwer erkrankt waren, auch nicht im Vergleich mit SARS-CoV-2-negativen Referenzpatienten. Bei ebenfalls gleicher Rate an stationären Aufnahmen hatten die COVID-19-positiven Patienten allerdings deutlich längere und kompliziertere Verläufe, inklusive eines beatmungspflichtigen Lungenversagens (ARDS). Auch nach Entlassung aus dem Krankenhaus zeigten die COVID-19-Patienten häufiger persistierende Symptome. Die ebenfalls hohe Hospitalisierungsrate bei erneuter Vorstellung zeigt, dass Ärztinnen und Ärzte der Notaufnahmen sorgfältig prüfen müssen, wen sie nach Hause entlassen können und wen nicht. Möglicherweise besteht hier ein Potenzial zur Verhinderung prolongierter Krankheitsverläufe durch den frühzeitigen Einsatz intensivierter Therapieverfahren. Hierzu fehlt bisher Evidenz für die spezifische Versorgungssituation in Deutschland, sodass *ReCovER* eine wichtige Datengrundlage für die optimierte Versorgung und die Identifikation von Risikopatienten in der ZNA schafft.

*ReCovER* ist ein Register mit hoher Granularität, dennoch ermöglicht der modulare Aufbau des Registers eine Dokumentation mit relativ geringem Aufwand. Neben der Charakterisierung des Krankheitsbilds und der Abbildung notfallmedizinischer Aspekte hinsichtlich der initialen Behandlung von COVID-19-Patienten ermöglicht die Plattform die Beantwortung weiterer wichtiger Fragen. So kann die Auswertung größerer Datensätze dazu beitragen, spezifische Verlaufsprädiktoren zu identifizieren oder aber die longitudinale Veränderung des Krankheitsgeschehens in Deutschland zu erfassen. Vor diesem Hintergrund ist es wünschenswert, dass sich zukünftig möglichst viele weitere Notaufnahmen an *ReCovER* beteiligen.

Zu Beginn der ersten Infektionswelle im Jahr 2020 bestand noch große Verunsicherung bezüglich der optimalen Strategie zum Umgang mit COVID-19 in den Notaufnahmen. Dagegen ist es im Lauf der letzten Monate durch die Steigerung der Testkapazitäten und weitere Maßnahmen wie Maskenpflicht und Lockdowns zur Entlastung von Notaufnahmen und Intensivstationen gekommen. Der Effekt der laufenden Impfung der Bevölkerung gegen SARS-CoV‑2 ist aktuell noch nicht zu beurteilen, wird aber voraussichtlich weitere Entlastung bringen. Dennoch sind – auch vor dem Hintergrund bereits weltweit nachgewiesener und teils hoch ansteckender Mutationen von SARS-CoV‑2 [2, 9] – weitere gemeinsame Anstrengungen erforderlich, um die Versorgung erkrankter Patienten in der Notaufnahme einerseits sowie den Schutz des dort tätigen Personals andererseits zu verbessern. Der longitudinale Vergleich mittels *ReCovER* ermöglicht zudem die Analyse der Inanspruchnahme medizinischer Ressourcen in verschiedenen Phasen der Pandemie. Zudem bietet die kooperative Forschung im Rahmen eines gemeinsamen Registers die Chance, wichtige Erkenntnisse für zukünftige Pandemien zu gewinnen.

## Limitationen und Stärken

Die aktuelle Auswertung hat mehrere Limitationen, die als registerimmanent bezeichnet werden können und sich vor allem aus der retrospektiven Erfassung von Routinedaten ergeben. Dies betrifft insbesondere fehlende Werte bedingt durch unterschiedliche Labor- und Diagnostikstandards zwischen den beteiligten Zentren. Die Aufnahme von Referenzpatienten in *ReCovER* beinhaltet die Gefahr eines Selektionsbias, wir versuchen dies über eine Auswahl nach der reinen zeitlichen Nähe der Vorstellung der Patienten in der Notaufnahme zu adressieren. Allerdings waren bei der vorliegenden Auswertung der ersten 1000 Patienten nicht die Daten aller eingeschlossenen Kontrollpatienten vollständig eingegeben. Dies erklärt die ungleiche Gruppengröße und mag zu einer gewissen Unschärfe beitragen. Zukünftige Auswertungen von größenäquivalenten Patientenkollektiven werden dies korrigieren. Prospektive Studien mit vergleichbaren Patientenzahlen – obwohl sicherlich von höherer Aussagekraft – sind aufgrund naheliegender logistischer und infrastruktureller Gründe (Kosten, erschwerte Einholung des Patienteneinverständnisses etc.) kaum in kurzer Zeit zu realisieren, sodass Register wie *ReCovER* eine wichtige Erkenntnisquelle darstellen. Insofern kann *ReCovER* auch als Blaupause für andere Fragestellungen in der Notfallmedizin dienen.

## Fazit für die Praxis

- Die aktuelle Pandemie stellt eine enorme Herausforderung für die Notaufnahmen dar. Unter dem Druck eines isolationspflichtigen Krankheitsbilds muss neben der Erstversorgung darüber entschieden werden, welche Teststrategien in der Notaufnahme schnell, kosteneffektiv und mit einer ausreichenden diagnostischen sowie prognostischen Wertigkeit angewendet werden können.
- Es herrscht weiterhin Unsicherheit über die Prädiktoren eines schweren Verlaufs von COVID-19, die in der Notaufnahme als Entscheidungshilfe dienen könnten. Register, wie die hier vorgestellte *ReCovER*-Studie sind geeignet, die erste Phase der klinischen Versorgung mit dem weiteren Verlauf zu verbinden.
- Standortübergreifende Register sind für den raschen Erkenntnisgewinn gerade im Fall von pandemischen Gefährdungslagen essenziell. Offene, angemessen granulare und krankheitsspezifische Register wie *ReCovER* eignen sich am besten, um schnell Informationen über medizinische Profile neuer Krankheitsentitäten zu erstellen und Handlungsempfehlungen abzuleiten.
- Mit *ReCovER* ist es gelungen, eine Plattform zu etablieren, an der sich mehrere große Universitätskliniken in Deutschland kontinuierlich erfolgreich beteiligen. Insofern kann *ReCovER* auch als Modell weiterer flächendeckender Registerprojekte verstanden werden.
